# Evaluating bias in forensic evidence: From expert analysis to AI-based decision tools

**DOI:** 10.1016/j.fsisyn.2025.100645

**Published:** 2025-11-06

**Authors:** Ido Hefetz

**Affiliations:** The Graduate Program in Science, Technology and Society , Bar-Ilan University, Ramat-Gan 5290002, Israel

**Keywords:** Forensic bias, Artificial intelligence, Expert analysis, Public engagement, Miscarriage of justice

## 1 Introduction

The Dreyfus Affair, a notorious scandal that unfolded in late 19th-century France, and the Brandon Mayfield case, which occurred in the early 21st century, both resonate in today's conversations about miscarriage of justice and the misuse of forensic expertise within law enforcement and criminal justice systems. At the heart of this scandal lie instances where forensic evidence was accepted uncritically and without a proper oversight or due process, leading to flawed adjudication: in Dreyfus's case, a French army officer was falsely convicted based on handwriting analysis distorted by antisemitic prejudice [1]; in Mayfield's case, a Muslim American attorney was erroneously identified through fingerprint analysis influenced by biases [2,3].

What is still disturbing, even after 130 years for Dreyfus, and more than two decades for Mayfield, is how science and emerging forensic techniques were misused to support prejudiced and despicable ideologies, and fuel public hysteria, demonstrating the dangers of overreliance on expert testimony when political, social, and military pressures distort judgment [1]. Not only did the Dreyfus Affair expose significant flaws in evidence assessment, but it also ignited a fervent public discourse on the interplay between justice and societal prejudices [1]. The Brandon Mayfield case highlights that even professional forensic experts are susceptible to cognitive biases, leading to critical errors in evidence interpretation [4,5].

These historical events raise crucial questions about the role of expertise in legal contexts, compelling us to reflect on how the lessons of the past continue to shape our understanding of justice today.

More than 130 years after Dreyfus and over 20 years after Mayfield, the integration of artificial intelligence (AI) into the criminal justice system presents both exciting advancements and significant challenges. Recent academic studies and practical applications in forensic science have increasingly begun to apply AI technologies more efficiently in areas that human experts lack the competence to excel. These include techniques, which raise concerns, such as gender differentiation through fingerprint analysis [6], predicting the number of contributors in DNA mixtures [7], and estimating shooting distance using network learning [8].

Although ongoing efforts aim to establish responsible AI guidelines, which emphasize human accountability and multi-level regulation [9], numerous investigations have shown that these systems can inherit and even amplify existing biases embedded in their training data or usage that lacks clear oversight and adjudication mechanisms, echoing the injustices seen in both the Dreyfus and Mayfield cases [10].

Furthermore, while modern forensic methods have evolved technologically, the underlying issues of bias and misinterpretation persist. This study asks: How do the biases inherent in human forensic expertise differ from those emerging in AI-driven forensic systems, and what lessons can be drawn to improve the reliability and accountability of forensic decision-making?

A crucial, but often overlooked, dimension of how experts and technologies interact is the *mode* of distributed cognition that the technology facilitates. I adopt and adapt a practical taxonomy proposed by Dror & Mnookin (2010) to analyze modes of human–technology interaction in forensic practice. The taxonomy distinguishes three modes: (1) *offloading*, where experts delegate routine or memory-intensive tasks to machines while retaining ultimate judgment; (2) *collaborative partnership*, where humans and algorithms jointly negotiate interpretation; and (3) *subservient use*, where humans defer to machine outputs and thereby suspend critical scrutiny. I use this taxonomy as an analytical lens to identify how different interaction modes produce distinct epistemic vulnerabilities and shape the formation of bias at the human–AI interface [3].

## 2 Epistemological foundations and methodological approach

The evolution of forensic science methods - especially in technology - since the Dreyfus Affair and the Mayfield case has been truly remarkable, with significant progress achieved across nearly all forensic disciplines. While earlier critiques argued that the interpretative role of forensic experts was under-examined [11,12], a growing empirical literature over the past decade has considerably expanded our understanding of cognitive and contextual sources of bias in forensic decision-making [[13], [14], [15]]. Contemporary studies document multiple, interacting sources of bias in forensic decision-making, including confirmation bias, where evidence is interpreted to support preexisting beliefs, and contextual bias, where extraneous case information affects judgments [[13], [14], [15], [16]].‏ Procedural mitigations such as blind verification and context management have been proposed and empirically evaluated to reduce these errors. Blind verification prevents one examiner's conclusions from influencing another, while context management limits exposure to irrelevant or potentially biasing information. Forensic evidence does not function as an isolated tool for identifying individuals or analyzing crime scenes; rather, it relies on the interpretation of forensic experts who draw on their extensive knowledge and years of experience. Crucially, however, expert interpretation is not produced in a vacuum. Recent theorizing on forensic cognition highlights that experts are embedded within a network of institutional practices, informational flows, and social pressures that can systematically shape judgments. Dror (2025) conceptualizes this dynamic as introduced at evidence collection, reporting, or investigative stages, that can propagate through coordinated layers of the justice system and amplify into decisive distortions. This perspective shifts our attention from individual expertise as an isolated source of truth to expertise as a node in a biasing ecology. Therefore, it demands analytical attention to those systemic interactions when we compare human and algorithmic sources of error [17].

The conclusions of experts, articulated in opinions submitted to the court and often supported by testimony, play a crucial role in the legal process by determining the appropriate weight given to forensic evidence. In recent decades, the notion of expertise has raised some questions, leading to increased scrutiny of forensic experts regarding their training and qualifications [17].

This paper is motivated by a central question at the intersection of epistemology and Science, Technology, and Society (STS) studies that influence the way we think of the use of expertise in judicial and policy processes: What is the difference between forensic expert bias and AI bias in criminal proceedings, and how do these biases contribute to wrongful convictions? This paper's central aim is comparative and analytic: I examine how epistemic vulnerabilities emerge in forensic practice by juxtaposing historical cases with contemporary instances of AI-supported forensic practice (e.g., automated matching and risk scoring). The goal is to use them as paired exemplars to test and refine an analytic framework.

Historically, the Dreyfus Affair reveals how expert testimony can be manipulated by political and cultural prejudices, while the Mayfield case shows how an innocent person's life can suddenly change following an undisputed expert's decision.

In modern contexts, AI-driven tools, while often perceived as objective, can inherit biases from the data on which they are trained and the opaque nature of their algorithms. The complexity and opacity of AI systems introduce additional barriers to understanding and contesting their conclusions [18]. For this reason, historical errors by human experts offer important insight into how we should evaluate and regulate new forms of algorithmic decision-making in forensic practice.

To explore this question, I adopt a qualitative, comparative case study methodology. This approach draws upon two distinct but interconnected case studies: the historical analysis of the Dreyfus Affair and the Mayfield case as exemplars of human expert bias, and contemporary examples as manifestations of algorithmic bias. I present an analytical framework grounded in three dimensions: bias, transparency, and accuracy. This will allow comparison of the epistemic vulnerabilities of human and machine-derived evidence. The comparison of forensic expert bias versus AI bias proceeds by asking, for each case, (a) which mode of human–machine interaction predominates (offloading, collaborative partnership, or subservient use); (b) how that predominant mode alters the provenance, amplification, and detectability of bias; and (c) which governance interventions (technical validation, workflow redesign, mandatory disclosure rules) are most appropriate given that mode [3].

## 3 The Dreyfus affair: a historical lens for expertise and AI in forensic science

In 1894, Alfred Dreyfus, a Jewish artillery officer in the French Army, was wrongfully convicted of treason for allegedly passing military secrets to Germany. While the case has far-reaching political and historical significance, this discussion focuses on its forensic dimensions. The conviction was based on questionable evidence, including a handwritten note, known as the bordereau, that was supposedly in Dreyfus's handwriting.

The note contained a list of documents related to French artillery. French intelligence officers quickly identified Dreyfus as the author. Alphonse Bertillon, the head of the Bureau of Identification in the Paris Police Department, conducted the key forensic analysis. He claimed that Dreyfus intentionally wrote the note to appear as if it were a forgery of his own handwriting, a tactic Bertillon called "self-forgery." Bertillon regarded his evidence as scientific, irrefutable, and conclusive, describing it as géométrique. In court, Bertillon testified as a handwriting analysis expert, leading the judges to adopt his conclusion.

The proceedings were closed to the public and were not transcribed. Sentenced to life imprisonment on Devil's Island, Dreyfus's conviction was fiercely defended by the military even as new evidence emerged. By 1896, Major Ferdinand Walsin Esterhazy was identified as the real spy during investigations by the Dreyfusards (supporters and advocates of Alfred Dreyfus), yet the military persisted in supporting the original conviction. The scandal gained widespread attention in 1898 when the French journalist Émile Zola's open letter, J'accuse … !, published on the front page of the newspaper L'Aurore, accused the French government and military of antisemitism and wrongful conviction. Ultimately, Alfred Dreyfus was retired from the French Army in 1899, having had his conviction upheld, albeit accompanied by a presidential pardon. It was not until 1906, following extensive public outcry and further investigations into the case, that he was completely exonerated and reinstated in his military position.

The Dreyfus Affair reveals the dangers of relying on a single expert within a politically charged legal system. It shows how expert opinion, when insulated from challenge, can facilitate institutional injustice [19].

The episode also highlights the enduring problem of misplaced trust in new forensic methods. Bertillon's system was respected in its time because it introduced what appeared to be a scientific technique. Today, similar beliefs surround AI-driven forensic tools, raising critical questions about the risks of misplaced trust in emerging technologies.

These parallels compel a reevaluation of how new forensic tools are introduced and assessed. For instance, imagine an alternate timeline in which every handwriting analysis underwent rigorous double-blind verification by independent interdisciplinary panels challenging such "evidence." Mandatory disclosure of expert backgrounds, systematic reviews by civil oversight bodies, and public commentary modeled on citizen science principles could have mitigated biases and prevented the miscarriage of justice that defined the Dreyfus Affair.

In the age of AI, this historical lesson remains urgent. The integration of machine-based systems into forensic practice must include mechanisms for scrutiny and contestation. Ensuring that both human and algorithmic interpretations are open to review is essential to maintaining the integrity of justice.

## 4 The progression of forensic evidence

Since the time of the Dreyfus Affair and the Brandon Mayfield case, forensic science has made significant strides. Improvements in forensic DNA recovery and analysis have positioned this field at the forefront of law enforcement efforts to combat crime [20]. Significant recent advancements include the application of massively parallel sequencing for analyzing STRs and other markers, also known as next-generation sequencing (NGS), which has significantly advanced DNA analysis in forensic science by providing more comprehensive and detailed genetic information than traditional methods [21,22]. Enhancements in DNA mixture interpretation through probabilistic genotyping methods have made it more reliable, objective, and defensible in court [23,24]. Profiling various RNA types to identify body fluids has provided a more precise method of determining the origin of biological samples at crime scenes [25]. Using SNP markers to predict forensically relevant phenotypes has added a powerful dimension to forensic science, allowing investigators to infer physical traits and ancestry from DNA samples [26]. Exploring epigenetics and DNA methylation to determine tissue types and estimate age [27], as well as the emerging field of forensic genetic genealogy, has contributed to solving cold cases and identifying previously unidentified human remains [28]. However, these advancements are not without challenges, particularly raising new ethical concerns about privacy and consent in the use of genetic data.

Recent progress in latent fingermark development and comparison has significantly enhanced forensic capabilities [29]. Key innovations include the use of advanced chemical reagents and imaging techniques, which have improved the visibility and detail of latent prints [30]. Techniques such as laser-induced fluorescence and digital imaging have enabled the detection of faint or degraded prints that were previously difficult to analyze [31]. Additionally, the integration of automated fingerprint identification systems (AFIS) with sophisticated algorithms has enhanced the accuracy and speed of fingerprint matching [32,33]. While these advancements increase the reliability of fingerprint evidence, they also address challenges related to partial or contaminated residue [34]. Yet, challenges remain, including the potential for false positives and the need for careful validation of new methods, as well as expert practice, to ensure their effectiveness and reliability in forensic applications [35].

These advancements also raise potential risks associated with using DNA and latent fingerprints as forensic evidence, emphasizing that reliance on such evidence is not foolproof. Instances of contamination, misinterpretation, and fraudulent practices have resulted in wrongful arrests and miscarriages of justice, underscoring the necessity for ongoing improvements and oversight in the field [36]. While the connection between forensic science and wrongful convictions has garnered increased attention over the past two decades, it remains a complex issue [37,38].

Additionally, while these safeguards might be developed for traditional methods, AI-driven forensic technologies lack equivalent oversight. Limited transparency, insufficient independent validation, and the absence of rigorous algorithmic audits risk perpetuating historical biases rather than mitigating them.

### 4.1 The Brandon Mayfield erroneous identification

On March 11, 2004, a terrorist attack in Madrid killed nearly 200 people and injured over a thousand. Investigators recovered partial fingerprints from a plastic bag linked to the attack and, after failing to match them locally, sent them to the FBI via Interpol. On March 19, the FBI's Automated Fingerprint Identification System (IAFIS) generated 20 candidates, and fingerprint number 17 was identified as belonging to American citizen Brandon Mayfield. Three FBI experts and a court-appointed examiner for the defense confirmed the match, leading to Mayfield's arrest on May 6.

However, Spanish police later identified the print as belonging to an Algerian immigrant, Onan Daoud. Mayfield was released on May 20, and the FBI retracted its findings after confirming Daoud's fingerprints. The Mayfield error reflected clear technical and procedural shortcomings that reinforce the broader argument of this article: miscarriages of justice often arise not only from individual bias but from systemic weaknesses in forensic workflows. The latent mark was partial and of limited quality, which made reliable comparison difficult [2]. IAFIS produced a candidate list, but examiners relied on that list without a fully independent, blind re-examination [2]. The Inspector General's review identified gaps in workflow, documentation, and verification procedures within the FBI [2]. Scholars have shown that such procedural weaknesses interact with confirmation effects to produce false positives [39]. Taken together, the limited material quality of the fingermark and the breakdowns in operational practice created conditions in which expert consensus reinforced a mistaken identification. The case exposed how cognitive biases, such as confirmation bias and circular reasoning, can influence forensic experts [40], and sparked widespread criticism of fingerprint analysis, highlighting the risks of unchecked expert authority and raising concerns about the reliability of forensic conclusions in court [41,42].

Both cases show the dangers of overreliance on expert testimony when political, social, and institutional pressures distort objective judgment. This aligns with Alan Irwin's critique of the traditional 'deficit model' of expertise, which assumes that only institutional scientists or forensic experts hold legitimate knowledge [43]. Both of these historical and modern contexts bring us to discuss that AI-based systems to be used in forensic domains must be scrutinized by the broader public to prevent miscarriages of justice.

## 5 Case studies of AI-induced misjudgments in the criminal justice system

The Dreyfus and Mayfield scandals warn us against uncritical trust in expert systems. The integration of AI into criminal justice processes is intended to enhance efficiency and objectivity, promising better predictions and unbiased decision-making [44]. However, as these tools have been deployed in real-world applications, critical flaws have surfaced, revealing that AI systems are far from infallible [45]. Misjudgments driven by biased data, flawed algorithms, and inadequate oversight have led to miscarriages of justice, often disproportionately affecting marginalized communities [46,47]. Here I explore several notable case studies where AI-induced errors have resulted in wrongful arrests, misidentifications, and biased risk assessments. These case studies have been selected to illustrate typical challenges in modern forensic AI systems to show how opaque algorithms and weak oversight echo the historical misjudgments of the Dreyfus Affair and Mayfield cases, and reignite familiar epistemic pitfalls.

### 5.1 Recidivism prediction

COMPAS (Correctional Offender Management Profiling for Alternative Sanctions) is an AI risk assessment tool used in the United States to evaluate the likelihood of recidivism among individuals in the criminal justice system. It uses a variety of factors to generate a risk score, including demographic information such as age, gender, and race, as well as details about an individual's criminal history, like previous arrests and convictions. Additionally, social factors such as employment status, education level, and family background are considered in the assessment. Based on these inputs, COMPAS produces a score that categorizes individuals into risk levels - low, medium, or high - indicating their likelihood of reoffending.

Flores et al. summarize several problems with the COMPAS risk assessment tool, highlighting issues of racial bias, accuracy, methodology, and applicability. One major concern is racial bias. It is argued that COMPAS is biased against Black defendants, overestimating their risk of recidivism compared to White defendants, raising concerns about fairness in the criminal justice system. Furthermore, the tool has been criticized for producing a higher rate of false positives among Black defendants (incorrectly predicting that a low-risk individual will reoffend) and false negatives among White defendants (incorrectly predicting that a high-risk individual will not reoffend) [48]. Methodological critiques have also been raised, such as those by Angwin et al., who suggest that certain analyses of COMPAS may impose a false dichotomy by oversimplifying risk categories, potentially misrepresenting the tool's predictive accuracy [49]. Last but not least, concerns about the inappropriate application of COMPAS have been noted, as the tool was designed for specific populations, such as probationers, and may not be valid for use with pretrial defendants, raising questions about its suitability outside its intended context.

Purves et al. claim that the use of AI tools like COMPAS in the criminal justice system poses significant risks due to issues of transparency [50]. Engel et al. argue that the manner in which COMPAS communicates its predictions to judges can obscure the extent of its biases, making it difficult for judges to fully understand how the algorithm's outputs might be skewed [51]. This lack of transparency can lead to decisions that are not fully informed or fair, thereby undermining the democratic legitimacy of judicial outcomes. When judges rely on predictions from AI tools without a clear understanding of the underlying biases, it compromises the fairness and accountability that are fundamental to the justice system.

Law enforcement authorities are driven to solve crimes, particularly serious ones. However, achieving a balance between predictive accuracy (efficiency) and fairness (equity) in risk assessment algorithms used in the criminal justice system remains challenging and elusive. Skeem discusses the inherent conflict between achieving high positive predictive value and minimizing false positive rates, particularly concerning racial disparities, and suggests that policymakers must decide which trade-off is more acceptable based on legal, ethical, and value considerations [52].

Thus, COMPAS mirrors the Dreyfus and Mayfield pattern: an illusion of scientific objectivity hides both algorithmic bias and the lack of external audit. This example not only illustrates the technical limitations of AI risk assessment tools but also highlights the urgent need for transparent evaluation of the training datasets and oversight mechanisms that are currently lacking.

### 5.2 Facial recognition misidentification

Facial recognition misidentification represents a critical challenge in the use of AI technology within the criminal justice system [53]. While these systems offer advancements in surveillance and security, they also pose significant risks of wrongful identification and bias, particularly against marginalized communities [54]. Kashmir Hill, a journalist at *The New York Times*, first reported the wrongful arrest of an innocent person due to the Detroit Police's use of an AI facial recognition system. She then discussed the broader issue of racism in law enforcement, emphasizing how the system falsely identified individuals from different cultural groups [55].

In 2020, Robert Williams, an African American man, was wrongfully arrested in Detroit after a facial recognition system mistakenly identified him as a suspect in a theft case. On a January afternoon, Robert Julian-Borchak Williams was at his office when he received a call from the Detroit Police Department instructing him to come to the station for arrest. Initially thinking it was a prank, he was shocked when, an hour later, police blocked his driveway in Farmington Hills, Michigan, and handcuffed him on his front lawn in front of his wife and daughters. The police showed him a photo with the words "felony warrant" and "larceny" but gave no further explanation.

Williams was taken to a detention center, where his mug shot, fingerprints, and DNA were recorded, and he was held overnight. The next day, detectives questioned him about a visit to the upscale Shinola store in Detroit's Midtown neighborhood, which had been robbed in 2018. Williams recalled visiting the store with his wife when it first opened in 2014. Williams spent 30 h in custody and faced significant emotional and reputational harm due to the wrongful arrest.

Facial recognition technology, often used by law enforcement agencies, has been shown to have higher error rates, particularly for people of color. A government report found that facial recognition algorithms exhibit higher false positive rates for people of color, especially Black and Asian individuals, across a variety of systems tested by NIST [56]. According to the NIST report, error rates were not uniform across all algorithms tested, but the most significant discrepancies were found in algorithms developed in the United States, where African Americans, Asians, and Native Americans were falsely identified more frequently than Whites. The false positive rates for African American and Asian faces were found to be 10 to 100 times higher than for White faces, depending on the specific algorithm [56].

One of the first studies to demonstrate how commercial AI systems misidentify women and people of color at significantly higher rates evaluated the performance of three commercial facial recognition systems from IBM, Microsoft, and Face++. It found significant racial and gender disparities in accuracy. The systems had error rates of 34.7 % for dark-skinned women, compared to 0.8 % for light-skinned men [57].

This case reveals critical failures in the use of AI-based forensic tools. The algorithm behind the facial recognition system was not transparent. Its decision-making process could not be independently reviewed or challenged. The misidentification also reflected a clear racial bias embedded in the system's design and deployment. These problems show how algorithmic tools can produce errors that are difficult to detect and correct.

Like the wrongful arrests of Alfred Dreyfus and Brandon Mayfield, this case demonstrates how unquestioned trust in algorithmic or human expert systems can lead to serious miscarriages of justice.

To avoid repeating the epistemic failures of past erroneous identifications, forensic science must confront not only the possibility of bias, but the fundamentally different ways in which it emerges and operates in human and machine-based decision-making.

### 5.3 Predictive policing tools

On January 11, 2022, an argument over household chores in a small apartment near Madrid turned violent when Lobna Hemid's husband, Bouthaer El Banaisati, attacked her with a broken piece of a wooden shoe rack. Hemid had previously reported enduring regular physical abuse and insults from el Banaisati. That night, as Hemid left the police station, officers used the VioGén algorithm to assess her risk, answering 35 yes/no questions. The system assessed her risk as "LOW", leading the police to provide no additional protection. El Banaisati was released the next day and, tragically, seven weeks later, he fatally stabbed Hemid before taking his own life.

Predictive policing tools, which use AI to forecast where crimes are likely to occur, have been criticized for reinforcing existing biases in policing [58]. The algorithm's failure to account for the complexities and volatility of such cases led to an inaccurate risk assessment. According to Hamilton, predictive policing tools often reduce complex human behaviors and situations to simplified numerical scores or categories. This oversimplification can ignore crucial contextual factors of individual cases, leading to inaccurate assessments [59]. In addition to bias and lack of transparency, predictive tools may struggle to account for the dynamic and unpredictable nature of human behavior and criminal activity. Their static models may fail to adapt to changes in social conditions or individual circumstances, resulting in outdated or inaccurate predictions.

The death of Lobna Hemid is an example of the need for caution and critical evaluation when deploying AI predictive tools in sensitive scenarios. It emphasizes the importance of rigorous validation, integration of human oversight, and consideration of contextual factors to improve the accuracy and effectiveness of predictive algorithms in ensuring safety.

These examples vividly highlight the consequences of inadequate oversight and the overreliance on static, opaque algorithms in high-stakes scenarios - a core concern of this research. In the context of this current investigation into forensic expert bias versus AI bias, these case studies highlight how the lack of rigorous, dynamic validation protocols can amplify inherent biases. In particular, the quality of training datasets is crucial; if these datasets embody historical prejudices or are unrepresentative, the resulting algorithms will perpetuate or even exacerbate these biases. Moreover, the absence of robust public and institutional review further entrenches these issues, as there is little external pressure to critically assess and update these systems. Ultimately, without the integration of comprehensive oversight mechanisms and continuous audits, both human and algorithmic decision-making remain vulnerable to bias, undermining the integrity of forensic evidence and the fairness of criminal proceedings.

## 6 Expertise in forensic sciences

The term "expert" is often seen as contentious by many scholars, not only because of its definition but also due to the practical reliance on the expert's knowledge in legal contexts. What makes one an expert, and more specific, what makes a trainee to become a forensic expert? In forensic science, expertise is not only a matter of reputation or authority, but a structured process of professional formation and validation [60]. Usually, it takes years to be recognized (by courts and professional bodies) as an expert. It develops through rigorous education, standardized training, and formal accreditation mechanisms that ensure both scientific competence and legal reliability [61].

Sheila Jasanoff explores the complex relationship between experts and society, critically examining their role in decision-making processes, particularly in science, technology, and policy. She concludes that expertise is not neutral or value-free and is intertwined with social and political contexts, and that experts bring their own biases, values, and assumptions to their work, which can influence their opinion, recommendations, and the resulting policies [62].

Stephen Turner critically examines the limitations and challenges of relying on expert knowledge, highlighting how experts' personal biases and values can influence their judgments. He advocates for a more critical stance by both policymakers and the public, emphasizing the importance of acknowledging the inherent boundaries of expertise [63]. In forensic science, this argument translates into institutional safeguards that ensure accountability, such as peer review, cross-examination in court, and independent auditing of laboratory practices, which constrain individual authority within transparent professional systems [64]. This perspective encourages the development of institutional mechanisms that ensure expert advice is transparent, accountable, and subject to public scrutiny, thereby preventing the undue concentration of power among experts and promoting a more democratic decision-making process [63]. Similarly, Rainer Grundmann investigates the difficulties experts face in modern knowledge societies. He argues that experts often struggle to convey their specialized knowledge to non-experts and to navigate the complex social and political environments in which their expertise is applied [65].

The definition and role of the expert have evolved over time, taking on different meanings in various historical contexts [66]. Historical methods and knowledge are increasingly being employed in legal proceedings to shed light on past events and aid in the pursuit of justice, providing a deeper historical perspective on the evolution of legal and scientific practices [67].

Yet, it is a difficult task to define "expertise" [68]. James Fleck's compilation of traits highlights the complexity of defining an expert. A non-exhaustive list of terms from recent literature on expertise includes: knowledge, know-how, competence, skill, information, structural repertoires, strategic recipes, hermeneutic continuum, professional knowledges, knowledge assets, intellectual property, paradigms, occupational and functional knowledges, cognitive constructs, and design hierarchies [69]. Each term depicts essential and unique aspects of expertise, yet the sheer number of concepts makes it challenging to discuss expertise as a unified category beyond its varied manifestations and specific details.

Even fundamental elements like "knowledge" and "experience" are debated when defining an "expert" [70]. While experience may be crucial for acquiring experience-based expertise, according to Collins and Evans, it is not sufficient on its own to define someone as an expert [71,72]. The dynamism of social life has prompted these sociologists to redefine what constitutes an "expert" and what qualifies as "expertise". To address these evolving concepts, they propose the establishment of a new field called Studies of Expertise and Experience (SEE), which aims to explore the boundaries of these terms. This innovative approach seeks to develop a normative theory of expertise that enables the evaluation of the quality of expert judgment. Understanding the epistemology of expertise involves philosophical inquiry into the underlying principles, methods, and processes through which experts acquire, justify, and validate knowledge in their respective domains.

Complementing these discussions, Alan Irwin [43] offers an alternative perspective by challenging the traditional, institutionally monopolized model of expertise. He advocates for a ‘citizen science’ approach - one that democratizes scientific authority by inviting laypersons and non‐expert reviewers to engage in the critique of forensic claims. Instead of deferring solely to the authority of forensic specialists, public institutions and independent reviewers, as Irwin suggests, should challenge and scrutinize the methodologies that underpin expert testimony. Recent policy analyses have echoed this argument, emphasizing that AI-based forensic systems must comply with the same principles of transparency, accountability, and independent verification that apply to human experts [73]. This perspective is especially relevant today in debates surrounding forensic AI, where the opacity of black‐box algorithms further underscores the need for collective examination. Transparency mechanisms such as open-source forensic AI models and explainability audits would empower not only forensic specialists but also legal professionals, journalists, advocators, especially civil rights advocates to question and validate forensic conclusions.

These integrated insights emphasize that expertise in forensic science is a complex, multifaceted construct, one that must continuously evolve to remain transparent, accountable, and democratically engaged in an ever-changing social landscape.

A promising example of how citizen science can be integrated into policy-making while balancing technical expertise with public engagement and oversight is the concept of exploratory regulatory sandboxes. These experimental environments allow stakeholders to collaboratively explore, test, and refine AI-driven policies before they are implemented on a larger scale. For instance, Kera and Kalvas [74] highlight how participatory regulatory experiments in an exploratory sandbox can prevent algorithmic decision-making from bypassing democratic deliberation, ensuring that policy frameworks align with societal values.

By involving diverse stakeholders, including legal experts, technologists, and citizen groups, such sandboxes foster transparency and accountability in AI governance.

Similarly, Cavallon and Kera [75] discuss the application of exploratory sandboxes in AI safety, particularly in the context of red-teaming AI systems for influence operations. Their work illustrates how involving external reviewers and non-expert participants can strengthen the reliability of AI-driven forensic tools and mitigate biases. These initiatives demonstrate that integrating participatory mechanisms into AI policy-making does not merely enhance technical robustness but also helps address ethical concerns regarding bias, accountability, and fairness in forensic applications.

## 7 Expert bias vs. AI bias

Both human examiners and AI algorithms introduce distinct but overlapping biases into forensic judgments. Understanding these differences is crucial for designing effective oversight. Itiel Dror has extensively analyzed how expert decision-making is influenced by contextual bias, cognitive shortcuts, and institutional pressures [40]. Dror illustrates the pervasive influence of cognitive biases in forensic decision-making, providing empirical evidence that reinforces the need for blind verification processes [17]. He demonstrates that small contextual biases introduced early in investigative workflows can "cascade" and "snowball", converting local interpretive errors into system-level distortions that affect human examiners' outputs. Distinguishing expert bias from AI bias requires tracing how each error propagates: human biases spread via institutional practices and informational cues, while AI biases are encoded in training data and model design but are amplified through the same socio-technical channels Dror describes [17]. The Brandon Mayfield case highlights how even well-experienced experts can be compromised by cognitive biases such as confirmation bias and overreliance on established patterns. Accepting Dror's diagnosis means that governance must interrupt not only individual errors but also the pathways through which small biases spread and grow [17].

However, it is important to recognize the limitations of much of the research on cognitive bias. Many studies, including Dror's, rely on dual-process theories, which divide human reasoning into fast, intuitive thinking and slow, deliberate thinking [76,77]. This framework can oversimplify the complex ways forensic experts integrate evidence and make decisions under uncertainty. An alternative perspective comes from probabilistic and Bayesian reasoning, which treats decision-making as a structured process of evaluating and updating evidence based on likelihoods [15,78]. In this framework, an examiner does not simply rely on intuition or patterns; instead, each piece of evidence is explicitly weighted, and beliefs are updated as new information becomes available.

While human forensic experts may develop bias through exposure to case details or organizational culture, AI systems encode bias at a different level: within their training data and algorithmic design. These systems often replicate historical inequalities and societal prejudices embedded in the data. Unless actively audited for fairness, such biases can go undetected and uncorrected [57]. In forensic science, predictive policing models and risk assessment tools such as COMPAS, as illustrated above, have been found to disproportionately classify minority groups as high-risk offenders [49]. This demonstrates that AI systems, while often perceived as neutral, can systematically reinforce historical injustices if their underlying biases remain unexamined.

Human bias stems from personal experience and social context. It is often visible in subjective reasoning and can be challenged through cross-examination or procedural safeguards. AI bias, by contrast, arises from patterns in training data and design decisions embedded in algorithms. These biases are harder to detect, especially when systems are proprietary or lack documentation. Without transparency and independent auditing, algorithmic errors may remain inaccessible to oversight and correction [79,80].

Furthermore, Eldridge et al. [81] demonstrate how an examiner's mindset in friction ridge comparisons can lead to errors. Their study provides concrete evidence that an examiner's internal expectations and cognitive frameworks, shaped primarily by professional culture and contextual influence, can significantly skew interpretations of fingerprint evidence. This finding is especially relevant in the context of both the Dreyfus and Mayfield cases. In Dreyfus, antisemitic bias distorted the interpretation of handwriting evidence. In Mayfield, a superficial similarity between fingermarks, combined with media pressure and a compromised approval process, contributed to a wrongful match. Eldridge et al.'s work, therefore, reinforces the argument that expert bias is not an isolated phenomenon but a systemic issue that continues to compromise forensic decision-making.

Because human and AI systems are vulnerable to different types of bias, forensic practice must include safeguards suited to each. Human judgment requires blind review and context management. AI systems need transparent design and independent validation. This approach should combine expert judgment with transparency in algorithmic tools. It must also remain accountable to legal processes and public oversight. This may involve implementing blind-review procedures for human examiners and encouraging greater transparency in AI tools through open-source auditing. Drawing from the Dreyfus Affair and the Mayfield case, we see that addressing bias is not merely a technical matter but also a philosophical and institutional one. From a philosophy of science perspective, the notion of complete objectivity in forensic practice may be unattainable. Instead, forensic science could aim for procedural objectivity, where systematic safeguards and transparency compensate for individual or institutional biases [82,83]. Recognizing the limits of human and machine reasoning encourages a reflective approach to evidence evaluation that strengthens judicial outcomes. Only through continuous reflection on the limitations of both human expertise and machine learning can we build a forensic system that aspires to fairness and justice [40,84].

Admittedly, decision-making in highly specialized fields such as forensic fingerprinting, where expert judgment is critical, differs from the development of AI-based systems by law enforcement agencies. In fingerprint analysis, the public's limited technical expertise may not effectively reduce errors. However, historical cases like the Dreyfus Affair and the Brandon Mayfield misidentification reveal that unchecked expert authority can lead to devastating miscarriages of justice. In both instances, entrenched institutional biases and overreliance on expert testimony contributed to wrongful convictions. In contrast, the development and deployment of AI systems can benefit greatly from the principles of citizen science. Citizen science refers to participatory mechanisms that involve non-specialists in the oversight of scientific and technical processes. In the forensic context, this could include lay reviewers, interdisciplinary panels, or civil society watchdogs who help monitor the development and application of AI systems. By involving diverse community members, interdisciplinary experts, and non-specialist stakeholders in the ethical oversight and review process, we can help counteract inherent biases and ensure that these technologies are applied in ways that protect individual rights. This participatory approach, informed by the lessons of both Dreyfus and Mayfield, fosters transparency and broadens accountability. It creates an additional layer of public scrutiny that may reduce errors before they lead to AI-driven miscarriages of justice.

## 8 Technoscientific futures and the power of prediction

AI-based predictive tools reflect how advances in data science and machine learning are reshaping the ways we manage risk and define justice. These tools are often designed to anticipate behavior and support decision-making in criminal justice processes. According to Neuwirth, such innovations increase the ability to influence human behavior through data-driven models [85]. However, this attempt to control future outcomes can produce unintended effects. When predictive systems rely on simplified or biased inputs, they may reinforce the very problems they aim to prevent.

This creates what Neuwirth describes as a paradox of control. The more we depend on algorithmic models to guide decisions, the less responsive those systems become to the complexity of real-world situations. In forensic and legal contexts, this may result in rigid, overconfident predictions that obscure important contextual details rather than reveal it. As a result, trust in such systems may weaken precisely because they fail to adapt to the unpredictability of human behavior and social context.

Despite their growing use, predictive policing tools are often adopted without standardized metrics to assess their accuracy or fairness. The lack of structured evaluation methods makes it difficult for agencies to distinguish between valid predictions and flawed ones, increasing the risk of poor policy decisions or unjust outcomes [86,87]. Academic references on the use of artificial intelligence systems in our daily lives are proliferating rapidly. It seems that the implementation of AI in criminal justice is imminent, and there is increasing awareness of the ethical considerations involved. This growing awareness aims to guide the responsible and effective integration of AI technologies within the legal field, ensuring their alignment with legal standards and societal values.

A good example of an academic reference to the integration of artificial intelligence in forensic practice is by Swofford and Champod, who argue that a best practice for implementing AI-based systems in forensic science is to adopt a gradual and responsible approach, from zero to full AI influence [88]. They propose a formal taxonomy of six levels of algorithm implementation, ranging from no algorithm influence (Level 0) to complete algorithm influence (Level 5). Yet, there is a risk that the nuances of human judgment and expertise may be overshadowed or lost, particularly in complex cases where contextual understanding is crucial.

Promoting education on AI security and raising awareness among practitioners and organizations about the risks associated with offensive and adversarial AI attacks, as well as strategies for mitigating these threats, is crucial in combating AI-driven misinformation and public opinion manipulation [89]. Combining knowledge-based AI research with data-driven AI provides a transparent and contestable framework, allowing for the cross-examination of AI systems' decision-making processes. This integration enables stakeholders to better understand how AI algorithms derive their conclusions and predictions, fostering accountability and encouraging collaborative discussions about ethical implications.

Combining empirical evidence with theory can reduce risks from biased AI outputs. This approach supports more trustworthy AI applications across fields [90]. However, mixing human expertise and AI may also introduce new biases from both sources. These combined biases can make it harder to identify and solve problems.

## 9 Have we learned from the Dreyfus Affair and the Mayfield case?

Reflecting on the Dreyfus Affair 130 years later and the Brandon Mayfield case more than two decades later, we must ask ourselves: have we learned from these historical injustices? While we have made significant advancements in forensic science and are exploring the potential of AI in criminal justice, the core lessons of these cases remain relevant. Just as anti-Semitic bias played a crucial role in Dreyfus's wrongful conviction, and cognitive bias contributed to Mayfield's misidentification, modern systems must guard against biases, whether human or algorithmic.

Trust in AI-based systems used in criminal justice depends not only on technical performance but also on their ability to support fair and accountable decision-making. Ananny and Crawford have argued that transparency is a necessary step toward accountability, but it is not a complete solution. In forensic settings, knowing how an algorithm operates does not guarantee that its effects on legal decisions will be understood or corrected [91]. It is important to note that different AI applications in forensic science present distinct challenges. Classification and detection tasks, such as fingerprint or DNA pattern recognition, generally rely on well-defined algorithmic processes, whereas interpretative functions, such as assessing complex forensic evidence or predicting criminal behavior, involve greater uncertainty and require careful integration of human judgment.

Richardson et al. emphasize that the data used to train predictive policing tools may already reflect patterns of civil rights violations. This introduces bias from the outset and allows it to influence outputs in ways that are difficult to detect or challenge [92]. These systems often lack meaningful opportunities for review. Unlike human experts, whose reasoning can be examined in court, algorithmic outcomes are rarely subject to direct questioning.

As Angwin and colleagues note, machine bias is not only a technical flaw. It reflects broader institutional and structural problems. Bias becomes more harmful when it cannot be challenged through legal or public mechanisms [49]. Building trust in forensic AI therefore requires more than transparency. It requires continuous testing, oversight by independent institutions, and clear procedures that allow biased outcomes to be contested.

Without these safeguards, algorithmic tools may reproduce the same errors that led to past miscarriages of justice, as seen in the Dreyfus and Mayfield cases.

The taxonomy of offloading, collaborative partnership, and subservient use also sharpens our understanding of the Dreyfus Affair itself. The court reliance on Bertillon's handwriting analysis was not simply a matter of flawed expertise, but it exemplified a subservient mode, where the authority of a technical claim overrode broader scrutiny. The "push of new evidence" in that case shows how deference to an apparently objective technique can displace critical evaluation. In contemporary settings, similar risks appear when algorithmic outputs are treated as decisive rather than advisory. Dror's framework shows that the devil lies not only in the quality of the evidence or the tool, but in the mode of interaction: whether experts' offload, collaborate, or defer. The examples depicted in this article show that the human-AI interface creates blind spots, where human bias may be concealed behind algorithmic authority, while algorithmic bias may be naturalized as objective expertise. Identifying which interaction mode predominates in each workflow is therefore critical for diagnosing bias and tailoring remedial governance.

For trust in AI tools to develop meaningfully within criminal justice, there must be a commitment to ethical principles and systematic evaluation before these tools are introduced into legal practice. There should also be a clear method for reviewing and challenging the outcomes they produce. Freiman discusses the difficulty of defining what makes AI trustworthy. He emphasizes the need to consider not only how algorithms function, but also the legal and ethical contexts in which they are applied [93].

This responsibility should not rest solely with developers or enforcement agencies. Broader participation is needed to ensure that AI systems reflect legal standards and social expectations [84]. AI systems can assist forensic professionals by automating routine tasks, and providing data-driven insights. However, the final interpretation and decision-making must remain under human control to ensure fairness, accountability, and adherence to legal standards. Otherwise, there is a risk that these tools will be seen as objective while operating without adequate accountability. The Dreyfus Affair and the Mayfield case both demonstrate what can happen when expert claims are accepted without critical review. In Dreyfus's case, Bertillon's flawed handwriting analysis was treated as reliable evidence, leading to a wrongful conviction without serious scrutiny.

Similar risks persist today. Algorithmic tools designed by technical experts may seem neutral and precise, yet their assumptions and biases are often hidden from public view. Like Bertillon's system, which projected a sense of scientific authority, forensic AI tools may be adopted based on their appearance of accuracy rather than careful validation. The gap between perceived reliability and actual fairness can become harmful when there are no clear procedures for public or legal challenge. When algorithmic processes are opaque, the promise of justice may give way to unexamined forms of bias [49,94].

Alan Irwin's research on public engagement reminds us that trust in technology must be earned. Systems that influence legal or forensic decisions should be designed to allow external scrutiny. The history of the Dreyfus Affair, and the role played by the Dreyfusards and journalists in exposing flaws, illustrates the importance of making expert authority contestable. The lesson remains relevant today. No form of expertise, whether human or machine, should be shielded from examination.

Concerns about delegating judgment to machines are not new. Hubert and Stuart Dreyfus raised these issues decades ago. In "Mind over Machine", they argued that human reasoning and intuition cannot easily be replaced by automated processes, especially in fields involving complex judgment [95].

This article has shown how the Dreyfus and Mayfield cases illustrate the risks of placing too much trust in unexamined expertise. Preventing wrongful convictions in these cases would have required stronger epistemic safeguards and institutions willing to revisit expert conclusions. In the context of AI, similar protections are needed. Transparent design, access to system data, and procedures for independent review can help detect and correct errors. The integration of AI into criminal justice demands more than technical innovation. It requires ongoing reflection and a firm commitment to ensuring that all sources of authority remain subject to critical review.

## 10 Conclusion

Historical and recent wrongful convictions emphasize the essential role of thorough scrutiny in evaluating human expert conclusions. Forensic science has seen considerable advancements in areas such as latent fingermark development, DNA analysis, and digital forensics. These developments enhance efficiency and accuracy, yet they introduce new challenges that must be carefully managed.

The increasing integration of artificial intelligence tools in forensic science holds the promise of improved precision and speed. However, these systems remain susceptible to biases and errors that can have significant and life-altering consequences. The influence of AI on forensic decision-making demands cautious and critical oversight.

Whether evidence is produced through established forensic methods or emerging technologies, it must always be treated with great care. The interpretation by forensic experts is pivotal to the justice process. Nonetheless, expertise is inherently limited. Expert opinions, although valuable, are not infallible. Overreliance on expert testimony, without recognition of its limitations and potential biases, can lead to grave errors.

The cases of Dreyfus and Mayfield highlight that both human expertise and AI systems are vulnerable to bias and misinterpretation. These cases emphasize the importance of continuous questioning and rigorous cross-examination of expert findings. This need becomes even more pressing as novel technologies such as AI are increasingly adopted in legal contexts.

Effective deployment of AI in forensic science must be guided by human oversight, adherence to rigorous standards, and a strong commitment to ethical principles. Failure to maintain such safeguards risks reproducing miscarriages of algorithmic justice. Transparency in methods and validation processes along with public accountability are essential to reduce these risks.

By respecting the epistemic boundaries of both human experts and AI-based tools, and through continuous reflection on their respective limitations, the justice system can reduce the likelihood of wrongful arrests and convictions. By respecting the epistemic boundaries of both human experts and AI-based tools, and through continuous reflection on their respective limitations, the justice system can reduce the likelihood of wrongful arrests and convictions. AI enhances forensic practice but does not replace the need for expert judgment and critical evaluation. Ultimately, these measures contribute to preserving the integrity and fairness of justice for all.

## Declaration of AI tools usage

During the preparation of this work the author used ChatGPT 3.5/4/5 to assist with spelling and grammar, and to help in rewriting sentences. After using this tool, the author reviewed and edited the content as needed and takes full responsibility for the content of the publication.

## Declaration of competing interest

The author declares that he has no known competing financial interests or personal relationships that could have appeared to influence the work reported in this article.
